# Resting State Connectivity Is Modulated by Motor Learning in Individuals After Stroke

**DOI:** 10.1177/15459683211006713

**Published:** 2021-04-07

**Authors:** Sarah N. Kraeutner, Cristina Rubino, Shie Rinat, Bimal Lakhani, Michael R. Borich, Katie P. Wadden, Lara A. Boyd

**Affiliations:** 1 University of British Columbia, Vancouver, British Columbia, Canada; 2 Emory University, Atlanta, GA, USA; 3 Memorial University of Newfoundland, St. John’s, Newfoundland, Canada

**Keywords:** motor learning, stroke, rs-fMRI, graph theory, functional connectivity

## Abstract

**Objective:**

Activity patterns across brain regions that can be characterized at rest (ie, resting-state functional connectivity [rsFC]) are disrupted after stroke and linked to impairments in motor function. While changes in rsFC are associated with motor recovery, it is not clear how rsFC is modulated by skilled motor practice used to promote recovery. The current study examined how rsFC is modulated by skilled motor practice after stroke and how changes in rsFC are linked to motor learning.

**Methods:**

Two groups of participants (individuals with stroke and age-matched controls) engaged in 4 weeks of skilled motor practice of a complex, gamified reaching task. Clinical assessments of motor function and impairment, and brain activity (via functional magnetic resonance imaging) were obtained before and after training.

**Results:**

While no differences in rsFC were observed in the control group, increased connectivity was observed in the sensorimotor network, linked to learning in the stroke group. Relative to healthy controls, a decrease in network efficiency was observed in the stroke group following training.

**Conclusions:**

Findings indicate that rsFC patterns related to learning observed after stroke reflect a shift toward a compensatory network configuration characterized by decreased network efficiency.

## Introduction

Damage resulting from stroke disrupts cortical networks and patterns of synchronized brain activity between disparate brain regions (termed functional connectivity).^1-3^ Synchronized patterns of brain activity can be characterized across the brain at rest (ie, resting-state functional connectivity [rsFC]) and their relationships represented as coherence. These patterns characterize functional reorganization of the brain after stroke and are reliable measure that characterize neural changes across the stages of recovery.^4^ While altered rsFC is associated with motor recovery (ie, improvements in function characterized by clinical assessments),^5^ it is not clear how rsFC is modulated by skilled motor practice after stroke (ie, behavioral improvements associated with a specific motor task). Even though rsFC does not rely on task performance, there is evidence showing that active networks mapped with rsFC overlap with regions involved in task performance.^6-8^ As rsFC does not rely on participant effort or compliance, it may be used to characterize neural changes that accompany motor impairment poststroke. Typically, in individuals with stroke, rsFC is disrupted in the sensorimotor network relative to healthy individuals.^9,10^ Increases in rsFC in both the sensorimotor network and between regions implicated in cognitive processes (ie, working memory) have been observed as motor recovery is achieved.^9,11,12^ For instance, poorly recovered individuals showed decreased connectivity within the sensorimotor network, while no differences in connectivity were observed between individuals who were well-recovered and healthy controls.^9^ Yet a typical pattern of connectivity is not necessarily restored during recovery after stroke. Even in well-recovered individuals, relative to healthy controls, reduced connectivity persists between brain regions associated with cognitive processes.^9,13^ To date, changes in rsFC have largely characterized functional reorganization that occurs in association with recovery from stroke.^11,12,14,15^ It remains unclear whether or not skilled motor practice drives changes in rsFC patterns.

Importantly, rsFC is thought to reflect the processing of information gained during skilled motor practice associated with motor consolidation and learning.^16,17^ Short-term changes in rsFC in areas previously shown to be critical to planning and executing visually guided movement^18,19^ including a network of frontal, posterior parietal, and cerebellar regions are associated with learning a visuomotor task.^16,17,20,21^ However, as behavioral change associated with task-specific learning plateaus, limited long-term changes in rsFC in healthy individuals are noted.^21^ While learning (and relearning) motor skills are critical to promoting functional recovery, we know little about the alterations in processes underlying motor learning after stroke. Thus, rsFC can be employed to characterize change in consolidation of motor memories and learning that result from functional reorganization after stroke.

Specifically, functional magnetic resonance imaging (fMRI) shows that healthy individuals shift brain activity from the prefrontal regions early in skilled motor practice to premotor cortical activation after learning occurs.^16,22^ This shift is not observed after stroke.^23^ The persistent and greater recruitment of frontal-parietal regions during motor tasks may reflect higher cognitive load during skilled motor practice after stroke.^24^ It also may be related to an overall decrease in network efficiency after stroke,^25,26^ that represents a lower overall capacity to transmit information and indicates that a compensatory network (ie, not restored to a neurotypical pattern of functioning) underlies motor processes.^23^ Taken together, alterations in consolidation and learning processes may arise after stroke, reflected by decreased network efficiency and greater reliance on cognitive processes during skilled motor practice. To test this idea, we probed (long-term) changes in rsFC induced by skilled motor practice to examine how brain reorganization supports learning after stroke.

The primary aim of the current study was to examine how rsFC is modulated by skilled motor practice after stroke. Furthermore, we sought explore how changes in rsFC are linked to motor learning. To address our objectives, we employed a between-group design whereby 2 groups of participants (individuals with stroke and age-matched controls) engaged in 4 weeks of skilled motor practice of a complex, gamified reaching task, that was designed to prevent early plateaus in performance. Clinical assessments of motor function and impairment, and brain activity were obtained before and after training.

We expected that rsFC would be differentially modulated from pre- to posttraining between groups. Because past work showed that individuals with stroke rely on prefrontal regions during skilled motor practice,^23,27,28^ and that greater recovery is linked to increased functional connectivity of frontal regions implicated in working memory,^9,24^ we expected to observe increased connectivity within the sensorimotor network, and between the sensorimotor network and prefrontal areas. In exploring the association between changes in rsFC and motor learning, we hypothesized that (1) improvements in motor behavior associated with task-specific learning would be related to decreased connectivity between regions implicated in working memory in individuals with stroke and (2) healthy controls would show minimal connectivity changes. Finally, we predicted that changes in network efficiency induced by skilled motor practice would occur differentially after stroke relative to healthy controls. Specifically, we predicted that healthy controls would show enhanced network efficiency that would reflect their increased capacity to transmit information. In contrast, we expected that individuals with stroke would show decreases in network efficiency reflecting a shift toward a compensatory network configuration to support learning.

## Methods

### Participants

Thirty-two individuals presenting with chronic (>6 months) stroke between the ages of 35 and 85 years and 31 age-matched controls were recruited for the study. Individuals were excluded if they (1) were unable to complete MRI scanning; (2) showed signs of dementia (<24 on the Montreal Cognitive Assessment)^29^; (3) had history of head trauma, seizure, psychiatric diagnosis, neurodegenerative disorder, substance abuse, or neurological or muscular deficits that affected task performance; or (4) could not engage in the motor task without arm or shoulder pain. The inclusion of a wide distribution of individuals with varied stroke severity was intentional, as the gamified motor task was designed to enable as many individuals as possible to complete. The institutional review board of the University of British Columbia approved the protocol, all participants gave written consent, and the experiment was conducted in accordance with the principles of the Declaration of Helsinki.

**Figure 1.**
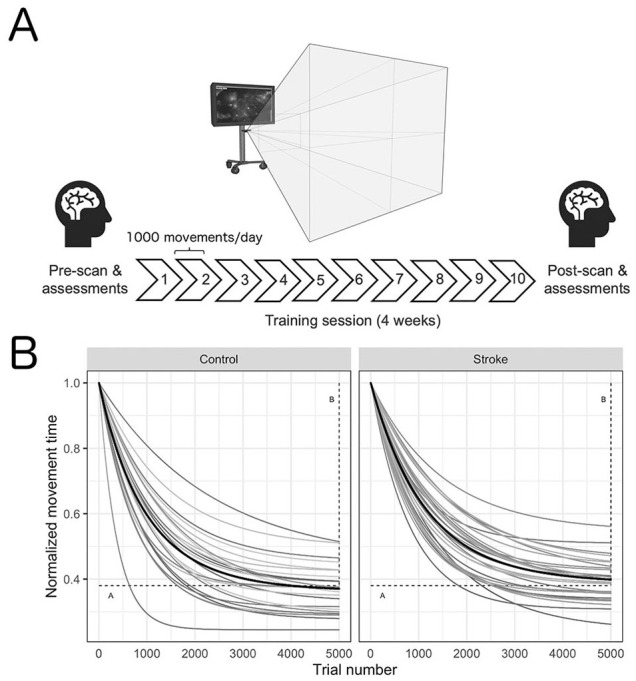
Timeline of the experimental design (A) and normalized acquisition curves for each individual participant and the group mean (black line) across all trials (B). Participants engaged in 4 weeks (10 total sessions, 10 000 total movements) of training on a gamified visuomotor reaching task using their affected arm (stroke) or nondominant arm (control). Within 24 hours prior to and following training, participants underwent functional magnetic resonance imaging to capture changes in resting-state functional connectivity. A decrease in movement time was observed for all participants during training. Motor skill acquisition parameters (A: the movement time at which the participant has plateaued in performance; horizontal; and B: overall change in movement time from the beginning of training to the point of performance plateau; vertical) are depicted by the dashed lines.

All participants engaged in 10 sessions of skilled motor practice (Figure 1). Participants completed structural and functional MRI scans no more than 24 hours (week 0; pretraining scan) prior to the first of 10 separate sessions and then a follow-up MRI acquisition session within 24 hours (posttraining scan; week 5) of the last session. Individuals in the stroke group also completed testing of motor impairment and function. Impairment was quantiﬁed using the upper extremity portion of the Fugl-Meyer (FM) Assessment (0-66; higher scores indicate less physical impairment).^30,31^ The Wolf Motor Function Test (WMFT).^32^ consisting of 15 timed movement tasks with higher scores reflecting a faster movement rate and thus greater function, characterized motor function before and after the training protocol (ie, administered within 24 hours prior to and completion of training). For each WMFT task, the rate (repetitions/60 second, with a rate of zero recorded if no repetitions were completed within 120 seconds) was calculated to characterize functional impairment.^33^ Trained physical therapists administered and scored the FM and WMFT.

### Behavioral Task

#### Apparatus

All participants engaged in a semi-immersive virtual reality–based intercept and release task (*Tra*ck and *I*ntercept *T*ask [TRAIT]).^34^ TRAIT was presented on a 46-inch monitor, viewed at 72 inches away (screen refresh rate 59 Hz). A Microsoft Kinect (Model No. 1517, Kinect for Windows; Microsoft) camera was used for motion detection and communication between the user and the computer task. Before each session, the Microsoft Kinect was calibrated using a 4-point grid; this enabled the workspace to be customized for each participant. All participants were asked to use their affected arm (stroke) or nondominant arm (control group) to navigate to each corner of the screen for calibration. All participants were asked to “save the world” by controlling an on-screen icon (spaceship) using movements of the relevant arm to intercept a moving object (asteroid) as it emerged from the side of the screen. Once intercepted, they had to throw the object to accurately hit a target (the sun), which caused the object to explode. The location of the target randomly varied on the screen. Auditory (ie, sound effects) and visual feedback (ie, a numerical score provided at the end of each trial) were used to maintain motivation and engagement in the task.

#### Procedure

Participants completed 10 TRAIT training sessions of increasing difficulty, which were designed to maintain challenge and to prevent plateaus in performance.^34,35^ Based on the principles of the challenge point hypothesis,^36^ the TRAIT sessions become more difficult as a higher degree of success was demonstrated by each individual. In this manner, each individual practiced in an optimally challenging environment given their own capacity.^37^ To advance to the next level of the task, participants had to achieve 80% success on 2 consecutive practice blocks.^34^ Difficulty was manipulated by increasing asteroid speed, decreasing asteroid size, and reducing target size. Each session lasted approximately 30 minutes and consisted of 5 consecutive TRAIT blocks. Each block contained 200 movements, totaling 1000 arm movements per session. In the experiment, each participant completed 10 000 total arm movements. The number of practice days and trials were determined from our prior work^34^ based on the principle that both repetition and intensity are important to stimulate experience-dependent neuroplastic change. Training took place 2 to 3 times per week for 4 weeks (Figure 1).

#### Data Analysis

Motor skill acquisition for the TRAIT sessions was quantified by exponentially fitting object interception time for each successful trial over the entire training period.^34,35,38^ Motor skill acquisition was quantified with 3 curve fitting variables (*A, B*, α). Briefly, the rate of skill acquisition, overall change rate of skill acquisition, movement time, overall change in movement time, and movement time at asymptote were extracted using the following:

$$E(MT_{N})\;=\;A\;+Be^{-\alpha N}$$

where *E*(*MT_N_*) is the expected value of movement time (*MT*) on trial *N. B* (seconds; our primary outcome measure) is a measure of overall change in movement time from the beginning of training to the point of performance plateau. Secondary outcome measures *A* (seconds) and α (seconds/trial) were used to identify the movement time at which the participant has plateaued in performance and quantify the rate of skill acquisition to the point of plateau, respectively.^37^ All values are reported as mean ± SD; for visualization values for each participant were normalized within each group.^35^

#### Group-Level Analyses

Separate 2-sample *t* tests were conducted on each outcome measure (*A, B*, α) to evaluate differences in motor performance between the stroke and healthy control groups. Data that did not meet the assumptions of normality were tested with nonparametric statistics to assess between-group differences for each outcome measure. All statistical analyses were conducted using R (R project for statistical computing) with an a priori α of *P* < .05 denoting significance.

### MRI

#### Data Acquisition

Magnetic resonance data were acquired at the University of British Columbia MRI Research Centre and were obtained on a Philips Achieva 3.0 Tesla whole body MRI scanner (Philips Healthcare), using an 8-channel sensitivity encoding head coil (SENSE factor = 2.4) and parallel imaging. The following scans were acquired: (1) 3D T_1_ turbo field echo scan (TI = 800 ms, short-TR = 1900 ms, flip angle θ = 6°, FOV = 256 × 256 mm, 160 slices, 1 mm slice thickness, scan time = 3.2 minutes), and (2) resting state blood-oxygen level dependent (BOLD) scans (2) with a single shot EPI sequence (TR = 2000 ms, TE = 30 ms, flip angle θ = 90°, voxel dimension = 3 mm^3^ with 1-mm gap, 36 slices, FOV 240 × 240mm, scan time = 8.0 min/scan). During the resting-state functional MRI scan, all participants were instructed to fixate on a picture of a window and not fall asleep.

#### fMRI Preprocessing and Analyses

All fMRI data were processed using the CONN toolbox with SPM (CONN v.17, Functional Connectivity SPM toolbox; McGovern Institute of Brain Research, Massachusetts Institute of Technology; www.nitrc.org/projects/conn, RRID:SCR_009550).^39^ Functional data were corrected for motion using the SPM12 realign and unwarp procedure with default settings, with any scans that exceeded 5.0 mm mean framewise displacement excluded from further analyses. Functional images were co-registered to their corresponding T1-weighted structural images. Using SPM12 unified segmentation and normalization procedure, structural images were normalized to SPM’s Montreal Neurological Institute 2-mm T1 template by an affine transformation and nonlinear registration, with the same estimated nonlinear transformation then applied to the functional data. Functional and anatomical data were then resampled into 2-mm and 1-mm isotropic voxels, respectively, and functional data were spatially smoothed with a Gaussian kernel (FMWH = 6 mm). To remove noise and lesion-induced artifacts, as this is critical to mitigate confounds associated with rsFC, particularly in stroke,^4^ data were decomposed into independent components at the group level to compute *Z*-scored spatial maps for each independent component.^40,41^ These spatial maps were thresholded (*Z* = 2.0) and noise components were identified via manual classification, according to the guidelines reported in Griffanti et al.^42^ To remove the effects of nuisance covariates, these identified noise components were then regressed out of the fMRI data, along with white matter signal and cerebrospinal fluid signal. The temporal signals in the 4-dimensional volume were linearly detrended and band-pass filtered (0.01-0.08 Hz) to remove undesired components.

The Harvard-Oxford probabilistic atlas was used to identify the regions of interest (ROI).^43^ In line with our research objectives, 31 ROIs were selected a priori for analyses, encompassing sensorimotor regions underlying motor function and learning (eg, primary motor cortex, supplementary motor area [SMA]; cerebellum; see Dayan and Cohen^16^ for review), dorsal attention stream which is critical for visuomotor and spatial processes (eg, angular gyrus of the posterior parietal cortex; superior parietal lobule),^18,44^ and key regions for cognitive processes (ie, working memory) implicated in stroke recovery (eg, thalamus, inferior temporal gyrus, superior frontal gyrus, parietal operculum).^5,45-47^ Functional connectivity correlation matrices (31 × 31; representing the level of functional connectivity between each pair of ROIs) were generated at the subject level. ROI-specific time courses of the BOLD signal were computed by averaging time courses across the voxels within each ROI. The resultant ROI-to-ROI correlation coefficients were Fisher *Z*-transformed and extracted to perform group-level analyses and brain-to-behavior correlation analyses.

To characterize network reorganization, we computed graph theory metrics at the subject level on resultant correlation matrices at each time point, after controlling for hand used in the task. Specifically, to characterize network distribution and interconnectedness, and to identify “hubs” (ie, highly connected ROIs), we extracted node degree (ie, the number of connections each node has with all other nodes in the network), betweenness centrality (ie, the influence that each node has over the transfer of information across nodes in the network, or the extent to which a node acts as a “hub”), and global efficiency (ie, the extent to which nodes of the network are integrated, representing the overall capacity that the network has to transfer information) for each participant^48-50^ using unweighted ROI networks thresholded at a cost value of *k* = 0.15.^39^ Resultant graph theory metrics were exported to text files from the CONN toolbox to perform group-level analyses using R. Importantly, these measures allowed us to quantify the network at each time point to further inform on any observed changes in rsFC at the group level.

#### Group-Level Analyses

To address our hypotheses, second-level analyses were performed in CONN.^39^ All analyses used a significance threshold of *P* < .05, FDR (false discovery rate) corrected for multiple comparisons (ie, correcting the individual connection-level statistics for the total number of individual connections in the ROI-ROI matrix).^39^ To account for lesion side, we included unaffected versus affected hemisphere (ie, training arm) as a covariate in all group-level analyses. Specifically, prior work has demonstrated that differences in connectivity may arise based on lesion side.^51^ Furthermore, functional differences in a left-lateralized versus right-lateralized system that supports motor function are well established in the literature, particularly for visually guided reaching tasks (encompassing sensorimotor and dorsal attention stream ROIs included in the current work; see Binkofski and Buxbaum^18^ for a review). Yet, as images are often flipped such that all lesions are analyzed within the same hemisphere, there are few motor learning investigations that consider these lateralized systems in individuals with stroke. Taking the above together, we opted to control for lesion side in analyses (ie, rather than transform images such that all lesions were within the same hemisphere) to permit the investigation of differences in connectivity within these lateralized systems regardless of lesion side. A Group (Stroke, Control) × Time Point (Pre, Post) ANOVA (analysis of variance) was conducted to examine overall differences in rsFC changes between groups. To determine how rsFC is modulated by skilled motor practice, within-group contrasts were conducted on the subject-level correlation matrices for each time point (posttraining scan vs pretraining scan), controlling for both hand used in the task (accounting for lesion side) and *B* value (ie, motor skill acquisition, our primary performance outcome). As hand used in the task is directly related to lesion side, this covariate effectively controls for lesion side in analyses. To test the association between motor learning and rsFC, a regression was conducted with *B* value as the predictor variable on changes in resultant connectivity (post > pre) within each group.

To quantify network characteristics and interconnectedness, betweenness centrality was calculated for each ROI for each participant and *t* tests against zero were performed for each ROI in CONN, separately for each group.^39^ Hubs were identified at the group level as ROIs that survived this calculation. Mean node degree for each ROI in the network was computed and reported for each hub, and the change in node degree (post minus pre) for each ROI separately for each group. Following the removal of an outlier in the stroke group that was equivalent to the mean plus 3 standard deviations of the mean global efficiency value, a Group (Stroke, Control) × Time Point (Pre, Post) ANOVA was computed on resultant global efficiency values to investigate differences in network efficiency across groups. Effect sizes were computed within each group comparing global efficiency values at each time point (using the average standard deviation of global efficiency values within each group) to characterize group differences. Lastly to consider the relationship between stroke-related demographics and changes in network efficiency, change in global efficiency values (Δefficiency; post minus pre) were correlated (via Pearson’s correlations) with time since stroke, and FM score.

## Results

Of the 63 participants, 28 (65.6 ± 11.3 years old, 8 female) individuals with stroke and 24 (64.0 ± 8.8 years old, 15 female, 22 right handed) controls were included in final analyses. Six control participants dropped out of the study (3 had incidental findings; 3 failed to complete the first scan). In addition, data from 4 individuals with stroke and 1 control participant were lost (for motion during scanning that exceeded our exclusion criteria of mean framewise displacement = 5.0 mm). Of the remaining participants, mean framewise displacement in the scanner was 2.1 ± 0.8 mm for the stroke group and 1.6 ± 0.6 mm for the controls. Demographics for each individual in the stroke group are reported in Table 1. The average level of the motor task achieved was 6 ± 2.9 (stroke) and 9 ± 1.8 (control).

**Table 1.** Demographic Information of the Stroke Group^a^.

Subject	Age	Sex	Time since stroke (months)	Fugl-Meyer (Upper Extremity) score	Wolf Motor Function Test (affected limb, reps/min)	Affected hemisphere	Lesion location
1	57	Female	162	32	11.9	Right	Middle frontal gyrus
2	51	Female	47	29	22.3	Right	Basal ganglia
3	58	Male	96	59	58.1	L	Inferior frontal gyrus
4	72	Male	49	50	38.7	Right	Basal ganglia
5	71	Male	117	59	68.9	Right	Middle frontal gyrus
6	77	Male	32	58	45.0	Left	Pons
7	47	Female	35	10	5.0	Right	Precentral gyrus
8	65	Male	46	33	22.0	Left	Parietal lobe
9	60	Male	188	31	21.0	Right	Parietal lobe
10	73	Male	60	54	38.3	Left	Thalamus
11	73	Male	30	–	34.6	Left	Lingual gyrus
12	58	Female	15	25	6.1	Left	Basal ganglia
13	66	Male	10	66	126.3	Right	Putamen
14	37	Female	84	18	21.0	Left	Basal ganglia
15	79	Male	27	54	40.8	Right	Basal ganglia
16	62	Male	8	59	74.5	Right	Putamen
17	78	Female	28	64	78.6	Right	Pons
18	72	Male	140	56	43.6	Right	Basal ganglia
19	75	Male	41	65	53.6	Left	Basal ganglia
20	79	Female	51	59	53.2	Right	Basal ganglia
21	59	Male	61	33	29.3	Left	Post central gyrus
22	61	Male	62	28	15.5	Left	Insular cortex
23	71	Female	94	52	43.1	Left	Basal ganglia
24	74	Female	19	39	32.5	Right	Precentral gyrus
25	51	Male	14	23	12.5	Right	Corticospinal tract
26	73	Male	47	64	51.9	Right	Thalamus
27	62	Male	6	62	37.4	Left	Amygdala
28	80	Male	135	58	34.5	Left	Precentral gyrus

a instances of missing data are indicated by a dashed line.

### Motor Skill Acquisition

An average of 84.7 ± 8.0% (stroke) and 86.34 ± 5.2% (controls) of the asteroids were successfully intercepted across all levels of task difficulty. Curve fitting revealed that all participants learned the motor task (positive *B* values) with resultant mean *B* values for the stroke and control of 0.61 ± 0.06 and 0.64 ± 0.07 seconds (see Figure 1 for acquisition curves across each participant). *A* and α values were 0.39 ± 0.06 (stroke) and 0.36 ± 0.07 seconds (controls), and 0.0009 ± 0.0002 (stroke) and 0.0011 ± 0.0004 seconds/trial (controls), respectively. Shapiro-Wilk and Bartlett’s tests showed data for each group met assumptions of normality, with the exception of α values. Thus, a Kruskal-Wallis rank sum test was conducted to analyze the between-group effect on α. No differences were observed between groups for our primary (*B* value; *t_B_*[47.49] = 1.44, *P* = .16) and secondary measures of learning (*t_A_*[47.49] = −1.44, *P* = .16; *H*_α_ = 2.88, *P* = .090).

### Rs-fMRI

**Figure 2.**
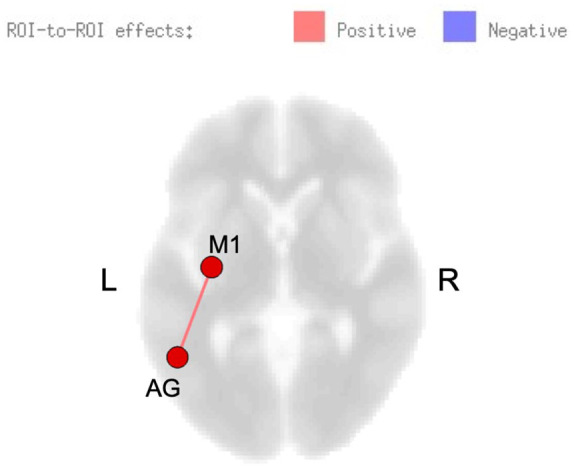
Resting-state functional connectivity (rsFC) modulated by skilled motor practice after stroke. Greater learning (*B* value) was positively correlated with connectivity between left precentral gyrus (M1) and left angular gyrus (AG).

A significant interaction between group and time point for rsFC was observed, showing increased connectivity between left superior lateral occipital cortex and sensorimotor regions (including SMA, premotor, and motor cortices), and decreased connectivity between right motor cortex and right superior parietal lobule for the healthy control group (post minus pre) relative to the stroke group. Within-group changes in rsFC (separately for each group) are summarized in Table 2. In the stroke group, within-group comparisons examining the effects of skilled motor practice on rsFC (accounting for *B* value) revealed enhanced connectivity within the sensorimotor network at the post versus pre scan between the following areas: left SMA and left supramarginal gyrus, left superior parietal lobule and right anterior inferior temporal gyrus, and right cerebellum Crus II and lobule III. Deceased connectivity was also observed between right cerebellum lobule III and left parietal operculum (Table 2). *B* value correlated with rsFC; positive correlations were noted between left angular gyrus and left precentral gyrus (Figure 2). In the control group, no changes were observed at the post- versus pre-scan, nor were any correlations found between connectivity and *B* value.

**Table 2.** Significant Functional Connectivity ROI-to-ROI Relationships Resulting From Group-Level Comparisons.

	ROI	Connected region	*t* value	*p*, FDR corrected
Group * Time point	R Precentral gyrus	R Superior parietal lobule	−3.12	.045
	L Superior lateral occipital cortex	R Supplementary motor area	3.02	.030
		R Superior parietal lobule	2.80	.043
		R Postcentral gyrus	3.31	.025
		R Precentral gyrus	3.22	.025
		L Superior parietal lobule	3.18	.025
Stroke				
Post > Pre	R Cerebellum III	L Parietal operculum	−4.62	.003
		R Cerebellum II	3.32	.041
	R Inferior temporal gyrus (anterior)	L Superior parietal lobule	4.47	.004
	L Supramarginal gyrus	L Supplementary motor area	3.74	.029
Effect of *B* value	L Precentral gyrus	L Angular gyrus	3.66	.035

Abbreviation: ROI, region of interest; FDR, false discovery rate; L, left; R, right.

### Network Characterization and Quantification

**Figure 3.**
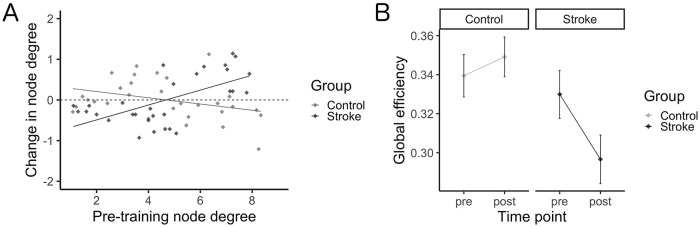
Network characterization. (A) Change in node degree (post minus pre) as a function of pretraining node degree. Across all ROIs in the network, node degree was observed to generally increase in the stroke group, while in contrast node degree was observed to generally decrease in the healthy control group. (B) Global efficiency across study time points for each group. Skilled motor practice was shown to induce a negligible change in global efficiency in the healthy control group, while in contrast decreases in global efficiency were observed in the stroke group.

**Figure 4.**
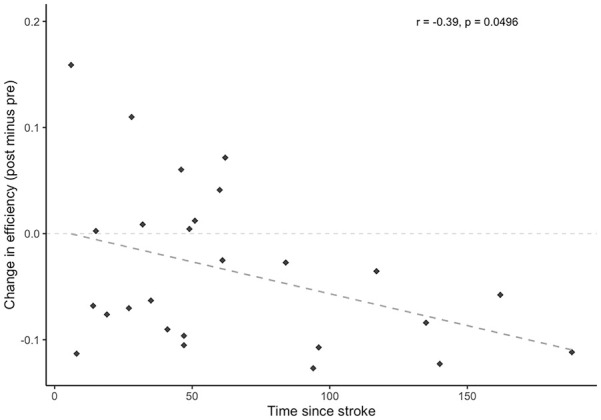
Change in global efficiency (post minus pre) within the stroke group as a function of time since stroke (months). Changes in global efficiency induced by skilled motor practice were shown to decrease with greater amounts of time that had passed since the occurrence of the stroke.

All identified hubs and their resultant node degree are included as Supplementary Materials (available online). Six hubs were identified in the control group at the pretraining scan, with 4 identified posttraining; 18 hubs were identified in the stroke group pretraining scan with 22 identified posttraining. The right middle frontal gyrus and right supramarginal gyrus were identified as hubs for both groups at each time point; additional hubs localized to frontal and parietal regions were identified in the stroke group including left middle frontal gyrus, left superior frontal gyrus, and bilateral angular gyri. Figure 3A shows the change in node degree for each ROI in the network, relative to its respective node degree at the pre-scan. A general increase in node degree across all ROIs in the network was observed in the stroke group, while a general decrease in node degree was observed in the healthy control group (Figure 3A). While no effect of time point was found (*F*[1, 49] = 2.1, *P* = .15), there was a significant main effect of group (*F*[1, 49] = 5.1, *P* = .029) and interaction between group and time point (*F*[1, 49] = 5.7, *P* = .021), whereby a decrease in global efficiency was observed in the stroke group (*d* = −0.52), while there was a negligible increase in global efficiency from pre to post in the control group (*d* = 0.19; Figure 3B). Pearson’s correlations indicates that Δefficiency correlated with time since stroke (*r*[24] = −0.39, *P* = .0496; Figure 4). No correlation resulted between Δefficiency and FM scores (*r*[24] = 0.04, *P* = .84).

## Discussion

The current study examined how rsFC is modulated by skilled motor practice after stroke. Consistent with our hypotheses, we observed increased connectivity in the sensorimotor network and circuitry underlying motor control, yet contrary to our hypotheses, we discovered an increase in connectivity within circuitry underlying higher cognitive processes. Coupled with the lack of differences observed in the control group, here we provide unique evidence that alterations in rsFC induced by skilled motor practice after stroke reflect a shift toward a compensatory network configuration, characterized by a decrease in network efficiency.

### Skilled Motor Practice Modulates Functional Connectivity After Stroke

Our data show changes in connectivity between regions previously implicated in cognitive load (eg, enhanced connectivity with anterior inferior temporal gyrus, decreased connectivity with parietal operculum) following training and underscore the notion that processes induced by skilled motor practice require greater cognitive load after stroke. Specifically, prior research demonstrated the role of inferior temporal cortices in working memory.^52^ The parietal operculum is implicated in stroke recovery due to its role in facilitating integration of proprioceptive feedback with preparation of subsequent movements, supporting optimal motor function^46^; decreased connectivity to this region thus indicates suboptimal motor function. Furthermore, our findings of enhanced connectivity following training in circuitry involving the SMA is consistent with prior work suggesting that after stroke tasks are experienced as more complex and require greater motor planning prior to movement execution.^24^ The SMA is a region critical for preparation of movement and transforming desired kinematics to forces exerted by the effectors.^53-55^ Thus, circuitry involving the SMA is heavily relied on during consolidation processes as revealed by associations between rsFC and recovery.^15,47^ Importantly, the semi-immersive virtual reality task employed here required the integration of visual, spatial, and proprioceptive information and was designed to challenge each participant throughout practice. Prior work demonstrated that both increased (cognitive) task demands and complexity require greater neural resources, affecting resultant patterns of brain activity.^56-58^ Furthermore, principles of learning and recovery, derived from simple motor (ie, sequence or finger-tapping) tasks, do not necessarily generalize to complex motor tasks.^59^ Thus, the complexity of the TRAIT in conjunction with its semi-immersive experience provides a unique opportunity to consider neural processes associated with motor learning and recovery poststroke.

Critically, the current study shows a unique pattern of rsFC patterns that support learning in individuals after stroke that was associated with connectivity between left primary motor cortex and left angular gyrus, regardless of hand used in the task (and thus lesion side). These regions are critical to the acquisition and performance of visually guided and object-based actions, with a left-lateralized system particularly critical to skilled, functional movements that are object related (eg, catching asteroids as in the virtual reality–based task employed here),^16,18,44^ and reflect the brain achieving a more effective state for motor learning.^60-62^ Yet, given that these changes differed from those seen in the control group,^3,13,23^ it is likely that this left-lateralized system is functioning in a compensatory manner. We may have observed different associations in our control participants because their rsFC was already optimized for motor learning. This idea is in line with prior work demonstrating that individuals with stroke show more benefit from consolidation processes relative to healthy controls.^60,61^ We did not include a no-intervention stroke group, as the window for spontaneous change related to upper limb function is early after stroke.^63^ Given that little spontaneous change is expected during the chronic stage of recovery from stroke, we conclude that alterations in rsFC observed in the current are attributable to task performance. In light of the current findings, the association between learning and rsFC after stroke likely reflects the reorganization of the brain to achieve a compensatory state that enables motor learning.

### Network Characterization

Our graph theory analyses showed that brain reorganization for motor learning after stroke comes at the cost of network efficiency and thus may represent a compensatory state.^23^ Typically, and consistent with the findings of our control group, as a network becomes functionally segregated 3 features emerge: (1) fewer ROIs act as hubs, (2) enhanced network efficiency results from high interconnectedness of hubs in the network, and (3) ROIs not critical to the specific function become less connected within the network.^12,48,50,64^ However, after stroke, we observed the opposite pattern of results. Following training, a general increase in node degree across all ROIs was observed in the stroke group, a large portion of ROIs were shown to act as hubs, and an overall decrease in network efficiency was observed relative to controls. Importantly, while prior work showed reduced efficiency after stroke relative to controls,^12,26^ here we demonstrate further reductions in efficiency with skilled motor practice, supporting the presence of a compensatory network underlying online and offline motor processes.^23^ Interestingly, changes in efficiency (post minus pre) were negatively correlated with time since stroke, further suggesting that network reorganization after stroke, and specifically later in the chronic phase, becomes robust and difficult to restore. While preliminary evidence demonstrated that high network efficiency after stroke is predictive of greater recovery outcomes,^65^ whether or not functional reorganization following a “window of recovery” may lead to enhanced (or restored) network efficiency in late stages of the chronic phase requires further research.

Additionally, it is necessary for future research to explore markers of recovery, through investigations examining the impact of a broad range of stroke-related factors on network efficiency. While on average our stroke group showed a decrease in efficiency with training, we identified 5 participants who showed (small) increases (ie, positive change over training). Prior research has shown that compensatory shoulder movements (ie, excessive abduction observed during clinical assessments) predict recovery outcomes.^66^ As the current study design did not permit an in-depth kinematic analysis, it is possible that participants performed movements during the task using different strategies (ie, engaging different groups of muscles resulting in compensatory movements). Furthermore, while we did not include prestroke handedness in our analyses as we were interested in capacity for change versus baseline motor ability, and an additionally conducted *t* test showed *B* value did not differ between prestroke handedness (right_*n*_ = 18 vs other_*n*_ = 10; *t*_15.28_ = −1.22, *P* = .24), how prestroke handedness may further affect strategies used during motor tasks and/or resultant motor learning. Thus, the extent to which compensatory movements may be linked to compensatory brain activity and/or prestroke handedness, and as such affected network efficiency should be addressed by future research. Other work demonstrated associations between the corticospinal tract integrity and recovery after stroke.^67^ While all participants in our study could perform the task, which might suggest they all had at least a partially intact, future work could consider the impact of descending white matter tract damage on network efficiency during motor learning.

It is important to consider timescales of learning and dose of training employed in the current study; individuals in the stroke group may have been in a different stage of learning as compared to the controls after the 4 weeks of training. It is possible that greater efficiency may have been achieved with a higher dose of practice; however, the unique features of the task used in this study may mitigate this concern. Importantly, we designed the TRAIT task following principles of the challenge point hypothesis.^36^ In the TRAIT task all participants make behavioral improvements from the start to the end of the study regardless of how quickly or to what level they advance. As the task is only advanced once a certain level of proficiency is achieved each participant moves up in difficulty according to their unique ability. This avoids the pitfalls that affect many typical motor learning studies where the motor tasks are relatively easy for healthy controls and at the same time very difficult for individuals with stroke. In the TRAIT task, the goal was for each participant to be engaged at their optimal level and not to reach plateaus in performance before completing the 10 000 practice trials. No between-group differences were observed in any of our behavioral outcomes suggesting that despite working at different levels of the TRAIT task both groups learned. In light of our findings, we stress that future studies of motor learning should consider the use of tasks that are based on the principles of individualized challenge.

While prior work demonstrated that frontal-parietal rsFC measured immediately prior to and following skilled motor practice of a tracking task is modulated by improvements in performance in healthy controls,^17^ we did not observe this pattern. Given that we know little about the differences between semi-immersive virtual reality and laboratory-based tasks, it may be that the absence of observable changes in rsFC in our control group is attributable to the point in time at which we measured rsFC (ie, 24 hours prior to and following training). Interestingly, our finding that there was not a pre to post difference in rsFC for controls is consistent with prior work employing a similar timeframe^21^ and may reflect that the network was already functioning efficiently. As we did not include an rsFC scan at a longer retention interval future research is required to understand whether or not changes in rsFC persist over time. Last, we did not flip images such that lesions were presented within the same hemisphere, but instead controlled for lesion side in our analyses. Our approach thus allowed us to detect changes in connectivity while taking functional differences within a left- versus right-lateralized system that supports motor function into consideration, regardless of lesion side. Because our study did not contain the power required to conduct further subgroup analyses to assess changes in connectivity between individuals with left versus right hemisphere lesions future research is required to investigate connectivity differences within these lateralized streams.

## Conclusion

In comparing rsFC before and after 4 weeks of skilled motor practice, the current study shows that changes in rsFC induced by skilled motor practice are altered after stroke relative to healthy controls. Here, we provide unique evidence of rsFC patterns related to learning after stroke, suggesting that patterns of change reflect a shift toward a compensatory network configuration characterized by decreased network efficiency. Future research should consider exploring biomarkers of recovery by including a more nuanced investigation of the impact of stroke-related factors network efficiency.

## Supplemental Material

sj-docx-1-nnr-10.1177_15459683211006713 – Supplemental material for Resting State Connectivity Is Modulated by Motor Learning in Individuals After Stroke Supplemental material, sj-docx-1-nnr-10.1177_15459683211006713 for Resting State Connectivity Is Modulated by Motor Learning in Individuals After Stroke by Sarah N. Kraeutner, Cristina Rubino, Shie Rinat, Bimal Lakhani, Michael R. Borich, Katie P. Wadden and Lara A. Boyd in Neurorehabilitation and Neural Repair
