# Supplementary material for: Resting State Connectivity Is Modulated by Motor Learning in Individuals After Stroke
Source: Neurorehabil Neural Repair. 2021 Apr 7;35(6):513–24. doi: 10.1177/15459683211006713 (PMC8135242; doi:10.1177/15459683211006713)
Supplement: sj-docx-1-nnr-10.1177_15459683211006713 – Supplemental material for Resting State Connectivity Is Modulated by Motor Learning in Individuals After Stroke [file sj-docx-1-nnr-10.1177_15459683211006713.docx]

Supplementary Table 1. Identified hubs for each group and time point

|  | Pre-training | | | | Post-training | | | |
| --- | --- | --- | --- | --- | --- | --- | --- | --- |
|  | Hub-region | *t*-stat | *p*-FDR corrected | Node degree (SD) | Hub-region | *t*-stat | *p*-FDR corrected | Node degree (SD) |
| *Stroke* | R Middle frontal gyrus | 2.82 | 0.027 | 4.8(2.5) | R Middle frontal gyrus | 3.43 | 0.007 | 4.1 (1.9) |
|  | R Inferior frontal gyrus (pars opercularis) | 2.30 | 0.050 | 4.1(2.1) | R Inferior frontal gyrus (pars opercularis) | 2.71 | 0.024 | 3.9(2.4) |
|  | R Superior frontal gyrus | 3.42 | 0.016 | 4.2(2.7) | R Superior frontal gyrus | 3.45 | 0.007 | 3.8(2.6) |
|  | R Postcentral gyrus | 3.66 | 0.011 | 7.0(2.0) | R Postcentral gyrus | 2.22 | 0.050 | 7.8(1.8) |
|  | R Precentral gyrus | 3.16 | 0.024 | 7.9(1.5) | R Precentral gyrus | 4.65 | 0.002 | 8.5(1.6) |
|  | R Supplementary motor area | 2.68 | 0.027 | 7.4(2.1) | R Supplementary motor area | 3.59 | 0.007 | 8.4(2.0) |
|  | R Angular gyrus | 2.78 | 0.027 | 4.2(1.9) | R Angular gyrus | 4.33 | 0.002 | 3.4(2.0) |
|  | R Supramarginal gyrus | 3.83 | 0.011 | 7.3(2.7) | R Supramarginal gyrus | 3.87 | 0.005 | 7.5(2.8) |
|  | R Superior parietal lobule | 3.09 | 0.023 | 7.0(2.3) | R Superior parietal lobule | 3.61 | 0.007 | 7.8(2.1) |
|  | R Superior lateral occipital cortex | 2.67 | 0.027 | 3.6(1.9) | R Superior lateral occipital cortex | 3.12 | 0.011 | 2.7(1.7) |
|  | R Thalamus | 2.47 | 0.036 | 1.7(1.7) | R Thalamus | 3.11 | 0.011 | 1.6(1.4) |
|  | L Middle frontal gyrus | 2.85 | 0.027 | 5.1(2.1) | R Parietal operculum | 2.22 | 0.050 | 5.4(2.7) |
|  | L Superior frontal gyrus | 2.82 | 0.027 | 4.6(1.5) | L Middle frontal gyrus | 3.19 | 0.011 | 4.3(1.8) |
|  | L Postcentral gyrus | 2.65 | 0.027 | 7.0(2.0) | L Inferior frontal gyrus (pars opercularis) | 2.52 | 0.031 | 3.5(2.0) |
|  | L Precentral gyrus | 3.67 | 0.011 | 7.8(1.8) | L Superior frontal gyrus | 4.45 | 0.002 | 4.3(2.3) |
|  | L Supplementary motor area | 2.68 | 0.027 | 6.1(1.8) | L Postcentral gyrus | 2.45 | 0.035 | 8.4(1.9) |
|  | L Angular gyrus | 2.56 | 0.031 | 4.6(1.7) | L Precentral gyrus | 3.42 | 0.007 | 8.0(2.0) |
|  | L Inferior temporal gyrus (anterior division) | 2.66 | 0.027 | 2.5(2.1) | L Supplementary motor area | 2.55 | 0.031 | 7.0(2.2) |
|  |  |  |  |  | L Angular gyrus | 2.23 | 0.050 | 3.9(2.2) |
|  |  |  |  |  | L Superior parietal lobule | 3.32 | 0.008 | 7.4(2.1) |
|  |  |  |  |  | L Superior lateral occipital cortex | 2.75 | 0.024 | 3.0(1.7) |
|  |  |  |  |  | L Cerebellum II | 2.58 | 0.031 | 2.9(2.2) |
| *Control* | R Middle frontal gyrus | 3.62 | 0.009 | 4.7(1.7) | R Middle frontal gyrus | 3.22 | 0.044 | 5.3(1.8) |
|  | R Inferior frontal gyrus (pars opercularis) | 3.01 | 0.027 | 3.5(1.8) | R Supplementary motor area | 3.20 | 0.044 | 7.5(2.0) |
|  | R Superior frontal gyrus | 4.14 | 0.004 | 4.5(1.5) | R Supramarginal gyrus | 3.08 | 0.044 | 6.2(2.0) |
|  | R Precentral gyrus | 3.19 | 0.021 | 8.3(1.4) | L Superior parietal lobule | 2.97 | 0.044 | 6.5(2.1) |
|  | R Supplementary motor area | 4.82 | 0.001 | 6.3(2.0) |  |  |  |  |
|  | R Supramarginal gyrus | 5.03 | 0.001 | 6.9(2.5) |  |  |  |  |
